# Laparoscopic resection of the hepatic caudate lobe: enhancing surgical precision with inferior vena cava-centric approach

**DOI:** 10.1093/jscr/rjag116

**Published:** 2026-03-07

**Authors:** Haoyang Huang, Dingwei Xu, Jie Huang

**Affiliations:** Department of Hepatobiliary and Pancreatic Surgery, The Second Affiliated Hospital of Kunming Medical University, Kunming 650101, China; Department of Hepatobiliary and Pancreatic Surgery, The Second Affiliated Hospital of Kunming Medical University, Kunming 650101, China; Department of Hepatobiliary and Pancreatic Surgery, The Second Affiliated Hospital of Kunming Medical University, Kunming 650101, China

**Keywords:** inferior vena cava (IVC) guidance, laparoscopic surgical technique, hepatectomy

## Abstract

Hepatic caudate lobe tumor resection is challenging due to anatomical depth. This study presents a laparoscopic technique for caudate hemangioma, using the inferior vena cava (IVC) as a guide to improve safety and feasibility. A 34-year-old male with incidentally detected caudate hemangioma underwent surgery. Preoperative evaluations (normal blood tests, ICG R15 3.0%, Child-Pugh A) confirmed good liver function. Computed tomography/magnetic resonance imaging confirmed the diagnosis, and surgery was planned with IVC dissection. The surgery lasted 191 minutes, with only 40 ml of blood loss and no intraoperative or postoperative complications. Histopathological examination confirmed a diagnosis of cavernous hemangioma. The patient achieved an uneventful recovery, with the drainage tube removed on postoperative Day 3 and hospital discharge completed on postoperative Day 4. Laparoscopic resection is safe for caudate hemangioma with proper preoperative assessment and experience. IVC guidance minimizes blood loss, shortens operative time, and ensures favourable recovery.

## Introduction

Hepatic haemangiomas are common benign liver tumours in adults, with an incidence of ~20% [1], predominantly affecting women aged 20–40 years and usually presenting as solitary lesions [2]. In contrast, hepatic caudate lobe hemangiomas are relatively rare. The caudate lobe has a unique anatomical location: it is deeply situated behind the three hepatic veins and hilar plate, and anterior to the inferior vena cava (IVC) [4]. This anatomical complexity makes caudate lobe hepatectomy a rare procedure, accounting for only 0.5%–4% of all liver surgeries [3]. This small but intricate hepatic segment receives dual blood supply from left and right portal veins as well as hepatic arteries, with venous drainage directly into the IVC via short hepatic veins. Resecting caudate lobe tumours is a major challenge in hepatobiliary surgery, largely due to the lobe’s complex and poorly understood anatomy, which also poses significant barriers to laparoscopic surgery. Experts have proposed several surgical approaches, including left-sided, right-sided, bilateral, and anterior transhepatic approaches, which traditionally involve splitting the liver along the middle hepatic fissure [5–6]. In our experience, the IVC serves as a critical anatomical landmark, guiding the surgical process like a lighthouse. Prioritizing the dissection and management of the IVC and its branches can improve surgical outcomes. This report, accompanied by a surgical video, details a case of laparoscopic caudate lobe hemangioma resection, with the aim of enhancing the safety of caudate lobectomy and reducing postoperative complications by elaborating on surgical techniques and key precautions.

## Case report

### Patients and methods

Approved by the Medical Ethics Committee of the corresponding hospital and compliant with relevant guidelines, this study enrolled a 34-year-old male with incidentally detected hepatic caudate lobe hemangioma (confirmed via computed tomography (CT)/magnetic resonance imaging (MRI), with normal laboratory tests). Surgical intervention was jointly decided with the patient after obtaining informed consent.

### Surgical techniques

#### Trocar and position

**Figure 1:**
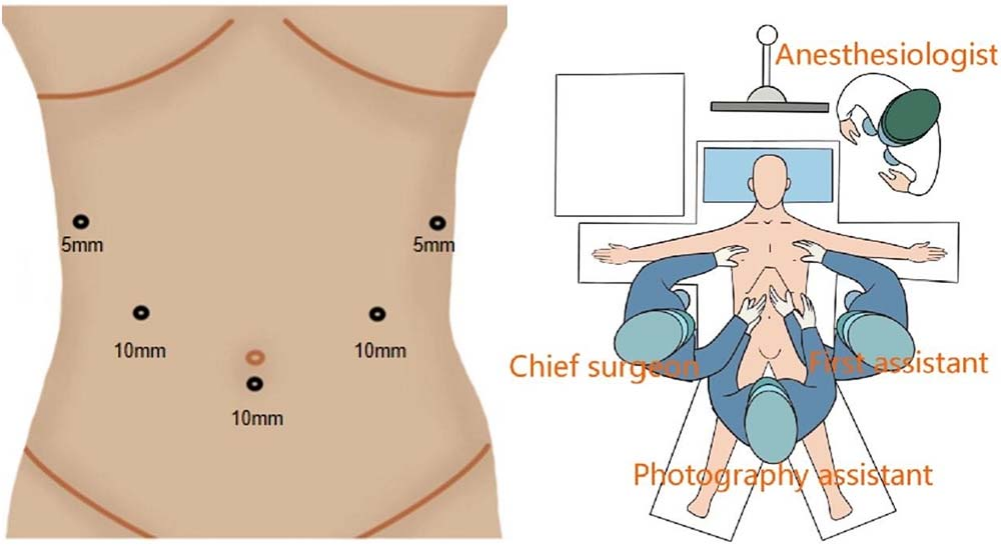
Trocar and position.

The patient was placed under general anaesthesia in a supine position with reverse Trendelenburg and right tilt. The legs were abducted, with the surgeon standing on the patient’s right, the first assistant on the left, and the camera assistant between the patient’s legs. Trocars were arranged in a U-shaped configuration (Fig. 1).

#### Dissection of the hepatic hilus

After dividing the falciform ligament, ultrasonic dissection of surrounding soft tissues exposed a roughly 7 cm hemangioma. Dissection of the liver’s inferior margin tissues was initiated from the right hepatorenal space to reveal the hepatic hilum, followed by the placement of a blocking band.

#### IVC and tumour exposure

**Figure 2:**
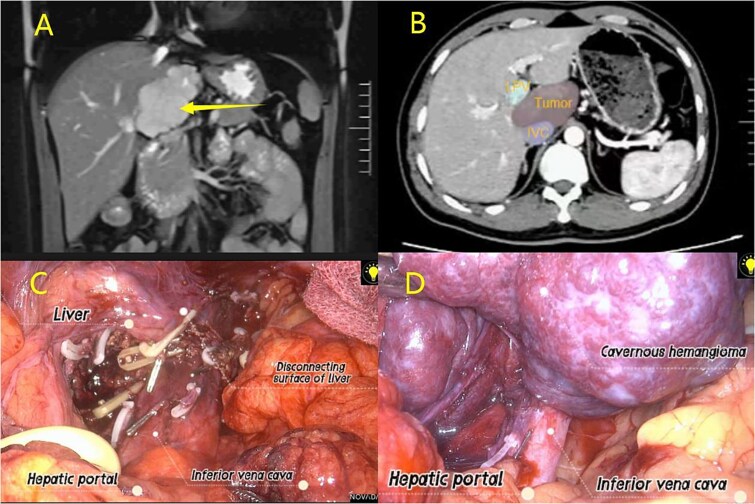
(A) MRI examination; (B) CT examination; (C–D) Laparoscopic resection of caudate lobe hemangioma procedure.

After hepatic portal blockage, dissecting tissues from the right hepatic hilum posteriorly exposed the IVC. The short hepatic veins between the IVC and caudate lobe were carefully isolated and secured with hemolocks and titanium clips. The left hepatic lobe was mobilized, the Arantius ligament dissected, G1L1 clamped with the same clips, and dissection finalized via ultrasonic scalpel. Cholecystectomy was additionally performed to enhance surgical visualization (Fig. 2; Video S1).

### Hepatic caudate lobe parenchymal resection

Guided by the IVC during hepatic parenchymal resection (especially with hepatic hilar occlusion), dissection began at the right hepatic hilum. An ultrasonic scalpel incised the liver along the hemangioma margin, following the IVC until the incision was perpendicular to it. The hemangioma was retracted left to fully expose short hepatic vessels between the caudate lobe and IVC; these vessels were carefully dissected, clamped, and transected. The ultrasonic scalpel continued to resect liver tissue until the lesion was fully freed. Bipolar electrocoagulation achieved hemostasis post-specimen removal, and a drainage tube was placed and secured in the surgical field (Fig. 2).

## Results

**Figure 3:**
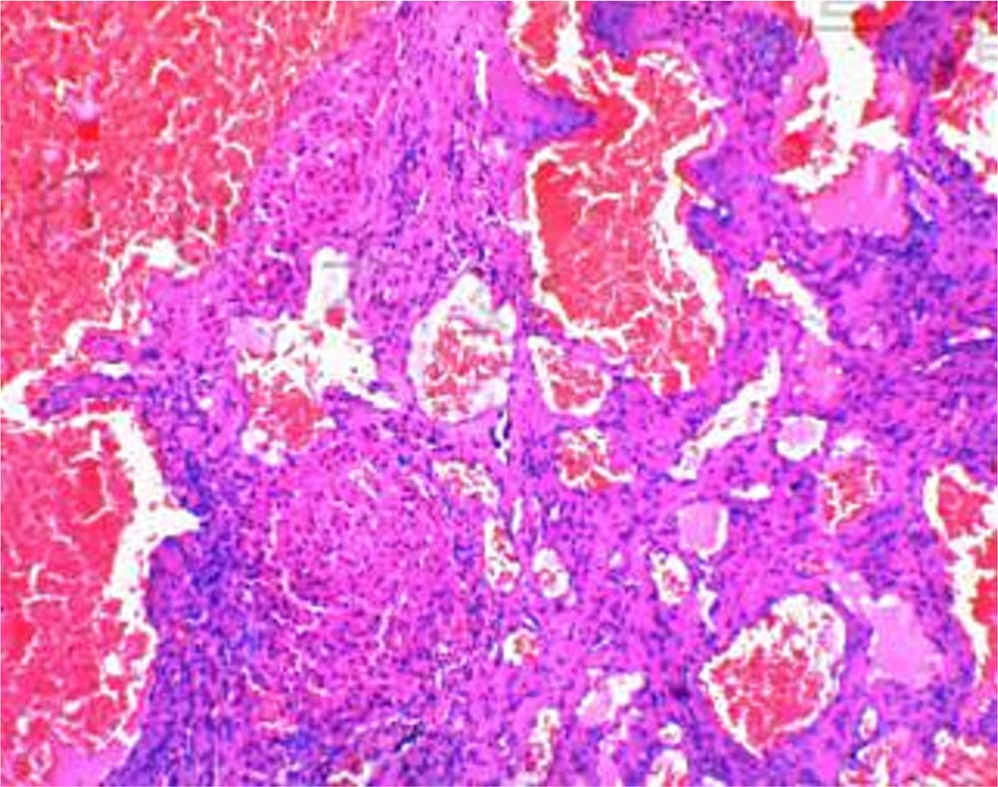
Pathological report.

The 191-minute procedure incurred only 40 ml of blood loss and no postoperative complications. Postoperative pathology confirmed hepatic cavernous hemangioma (Fig. 3). The drainage tube was removed on postoperative Day 3, and the patient was discharged on Day 4.

## Discussion

Surgical intervention is currently the preferred treatment for hepatic hemangiomas >4 cm in diameter, especially those causing upper abdominal discomfort or pain [7]. However, laparoscopic caudate lobectomy requires exceptional surgical expertise, relying on the operator’s extensive experience in liver resection, comprehensive understanding of hepatic anatomy, sound surgical judgment, and advanced laparoscopic skills.

A key challenge in laparoscopic caudate lobectomy is achieving adequate exposure. Full visualization of the caudate lobe, hepatic hilum, and associated vasculature is essential to minimize intraoperative bleeding and ensure procedural safety, and cholecystectomy may be strategically performed to improve surgical access in some cases.

The caudate lobe’s venous drainage directly into the IVC via short hepatic veins creates a high risk of uncontrollable hemorrhage if dissection is not performed with extreme care, especially given the greater number of short hepatic veins on the left side. Prioritizing the management of the IVC and its branches not only facilitates exposure but also reduces bleeding risk. We emphasize the critical role of the IVC in laparoscopic caudate lobectomy, as its strategic management can significantly improve surgical navigation and visual field.

The caudate lobe has a complex vascular supply: the Spiegel lobe and paracaval portion are mainly supplied by the left portal vein branch, while the caudate process is fed by the right portal vein branch [8]. Its arterial supply typically includes two vessels—one from the left or middle hepatic artery to the Spiegel lobe and paracaval portion, and another from the right hepatic artery to the caudate process [9]. Occluding these vessels can shrink and soften the hemangioma, optimizing the surgical field.

The Pringle maneuver (hepatic portal occlusion) is crucial for reducing intraoperative bleeding. We recommend a 15-minute occlusion period followed by a 5-minute release to minimize ischemia–reperfusion injury and intestinal congestion, with occlusion duration not exceeding 20 minutes to avoid liver damage.

Hepatic caudate lobectomy remains underreported due to the rarity of caudate hemangiomas and the procedure’s inherent risks. This report aims to enrich the body of knowledge on caudate lobectomy, reduce surgical risks and postoperative complications, and ultimately improve patient outcomes.

## Conclusions

Prioritizing the IVC as an anatomical landmark in laparoscopic caudate lobectomy is crucial. This approach not only minimizes intraoperative bleeding but also facilitates tumor exposure, thereby improving the safety and feasibility of the procedure.

## Supplementary Material

VIDEO_x264_rjag116
